# Gastrointestinal manifestations in Satoyoshi syndrome: a systematic review

**DOI:** 10.1186/s13023-020-01395-8

**Published:** 2020-05-19

**Authors:** Julián Solís-García del Pozo, Carlos de Cabo, Javier Solera

**Affiliations:** 1 Department of Internal Medicine, Complejo Hospitalario Universitario de Albacete, Albacete, Spain; 2 Department of Medical Sciences, Faculty of Medicine, Universidad de Castilla – La Mancha, Albacete, Spain; 3 Research Department, Neuropsychopharmacology Unit, Complejo Hospitalario Universitario de Albacete, Albacete, Spain; 4 Hospital General Universitario de Albacete, Unidad de Neuropsicofarmacología, Edificio de Investigación, 3ª planta c/ Hermanos Falcó, 37 E-02008, Albacete, Spain

**Keywords:** Diarrhea, Malabsorption, Rare diseases, Satoyoshi syndrome

## Abstract

**Background:**

Satoyoshi syndrome (SS) [OMIM 600705; ORFHA 3130] is a multisystemic disease with a probable autoimmune basis, whose main symptoms are muscle spasms, alopecia, diarrhea and skeletal alterations. Chronic diarrhea may be severe and result in malnutrition, anemia, growth retardation, cachexia, disability and even death. However, to date, no review of the digestive symptoms has been carried out.

**Methods:**

A search was performed in MEDLINE, Scopus and Web of Science databases. Cases of SS, without language or date restrictions, were recorded. Sixty-seven cases of SS were found up until December 2019. Thirty-nine cases described gastrointestinal manifestations.

**Results:**

Chronic diarrhea was the main digestive symptom (92.3%). Other symptoms such as abdominal pain (15.4%), nausea (7.7%) and vomiting (7.7%), were less frequent. The D-xylose test was positive in 10 out of 12 patients, and 9 out of 13 cases showed a flattened oral glucose tolerance test suggesting carbohydrate malabsorption. Antinuclear antibodies were detected in 8 out of 16 cases. Antibodies to stomach or duodenum tissue lysates were also detected by Western blot. Histological data revealed predominantly lymphoplasmacytic inflammatory infiltrate that can affect any section of the digestive tract. In 6 out of 10 patients, diarrhea improved with a treatment regimen that included corticosteroids. Other treatments, such as methotrexate, carbohydrate restricted diets or otilonium bromide, improved digestive symptoms in isolated patients. Improvement of symptoms up to three years of follow-up has been described. None of the three patients who died had received corticosteroids or immunosuppressants.

**Conclusion:**

Chronic diarrhea with malabsorption is one of the most disabling symptoms in SS. The early recognition of this disease is essential for immunosuppressive treatment and a better outcome.

## Introduction

Satoyoshi syndrome (SS) [OMIM 600705; ORFHA 3130] is a multisystemic disease, characterized by muscle manifestations in the form of painful spasms or cramps, diarrhea, alopecia, skeletal alterations, growth retardation and menstrual abnormalities [1]. The association of this syndrome with other autoimmune pathologies, the detection of autoantibodies in these patients and their response to immunosuppressive treatment, has led to postulate its autoimmune origin [2, 3].

Among the most typical features of the syndrome is diarrhea [4]. Chronic diarrhea and malabsorption can lead to malnutrition, weight loss, growth retardation [2], iron- deficiency anemia [5] or hypoproteinemia [6]. Untreated diarrhea has been described as one of the leading causes of morbidity and mortality in patients with SS [1].Diarrhea with signs of malabsorption, weight loss or growth retardation, and the detection of autoantibodies, are present in SS and other diseases such as celiac disease, tropical sprue or autoimmune enteropathy.

Previous reviews of SS have focused on muscle symptoms and alopecia [7–10] Our objective in this review is to offer an updated view of the gastrointestinal manifestations and their treatment response in SS.

## Main text

### Material and methods

#### Search

**Fig. 1:**
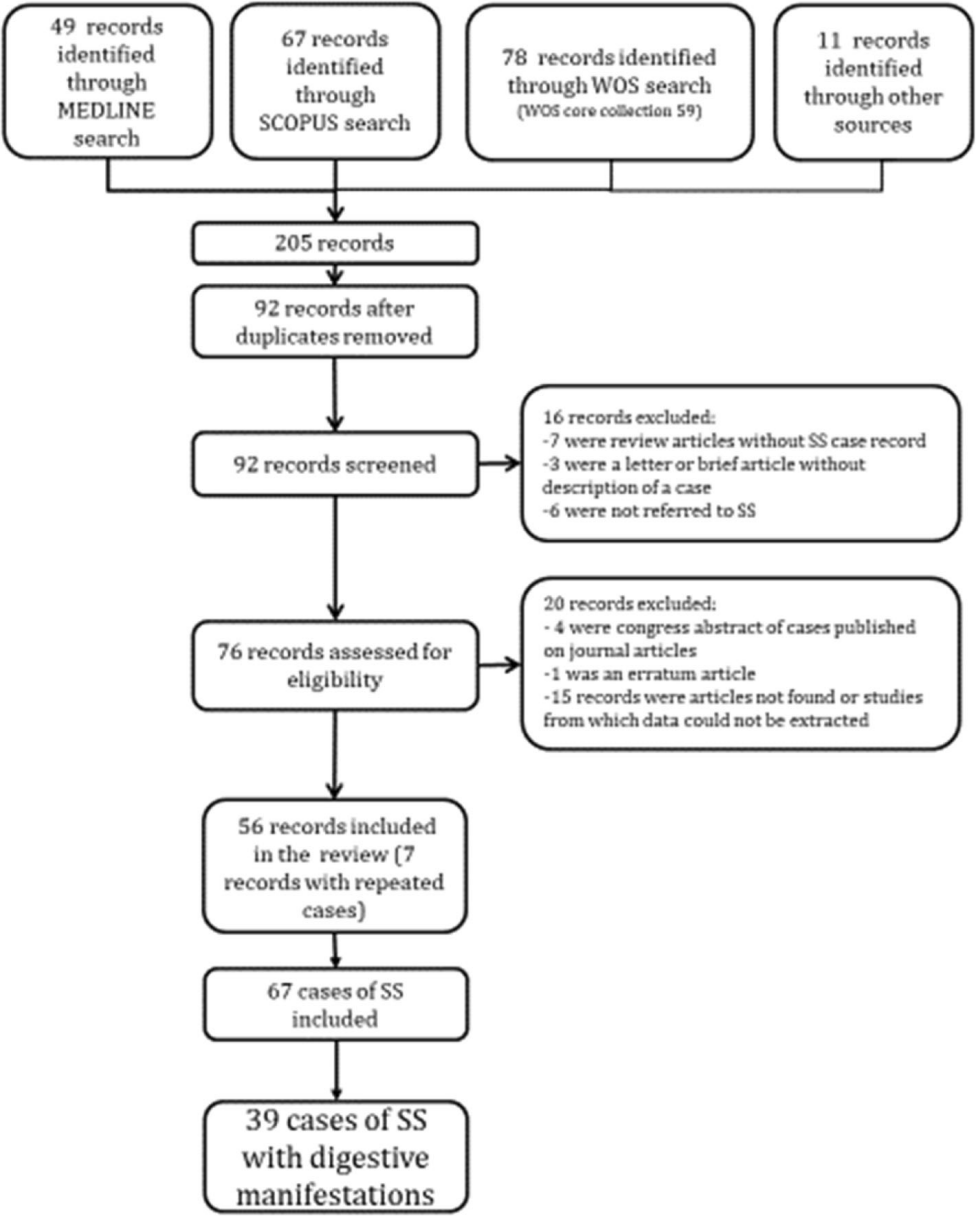
Flow chart illustrating case selection strategy

All cases were searched in MEDLINE, using the search strategy: (“Satoyoshi syndrome” [Supplementary Concept] or “Satoyoshi syndrome” [All Fields], or “komuragaeri disease” [All Fields]) or Satoyoshi [TI]). We also searched in Scopus and the Web of Science, using the keywords “Satoyoshi”, “Satoyoshi syndrome” or “komuragaeri disease”. We included all cases up to December 2019, without limiting year of publication or language. We also explored the references of the OMIM, ORPHANET and Rare diseases web pages. The lists of references from the articles found by electronic search were also reviewed to identify additional records. The results of the search are shown in the flow chart in Fig. 1.

A total of 56 publications describing 67 cases of SS were found; 39 (58.2%) cases described digestive manifestations [1, 2, 4–8, 11–32] (Table 1). The cases that were described across several publications were counted as one single case, and the data provided by the additional publications were pooled together to complete their description [33–37].

**Table 1:** Clinical characteristics, treatment and outcome in 39 patients with SS and digestive manifestations

	Author (year) [ref]	Age of onset (age of onset digestive manifestations)	Sex	Malabsorption tests	Autoantibodies	Endoscopy	Histology	Treatment	Outcome
**1**	Bledsoe I (2019) [32]	11	Female	NR	NR	NR	NR	Steroids, IVIG, plasmapheresis, methotrexate, and tacrolimus. Botulinum toxin.	NR
**2**	Al Dallal R (2019) [31]	12 (12)	Female	NR	NR	NR	NR	IVIG and systemic corticosteroid therapy. Rituximab, carbamazepine, imodium.	NR
**3**	Solera J (2017) [11]	10 (12)	Female	Carbohydrate malabsorption/D-xylose.	Anti-ACh receptor. Anti-GAD.	NR	NR	Carbamazepine and gabapentin, IVIG, prednisone, methotrexate.	Resolution of diarrhea with methotrexate and corticosteroids.
**4**	Li J (2017) [12]	6.5	Female	NR	NR	NR	NR	Prednisone, carbamazepine.	Improvement of diarrhea.
**5**	Aghoram R (2016) [13]	28 (30)	Male	NR	Anti-ACh receptor. Reactivity to unknown neuronal antigens.	Sigmoidoscopy revealed inflamed colon.	Collagenous colitis.	Steroids and mycophenolate mofetil, phenytoin, IVIG, plasmapheresis.	Improvement of diarrhea.
**6**	Rudnicka L (2014) [7]	36 (37)	Female	NR	ANA (1/640), anti-La/SSB, anti-histone antibodies, trace of dsDNA.	NR	NR	Triamcinolone and phenytoin.	NR
**7**		4 (4)	Female	NR	ANA (1/160)	NR	NR	Prednisone, cyclosporine.	NR
**8**		1 (1)	Female	NR	NR	NR	NR	Prednisone, ciclosporin.	NR
**9**	Merino de Paz N (2014) [14]	4 (4)	Female	NR	Anti-gliadin	NR	NR	Carbamazepine, otilonium bromide.	Disappearance of diarrhea.
**10**	Rosales RL (2013) [15]	0 (0)	Female	NR	NR	Esophagogastroduodenoscopy and colonoscopy. Flattened mucosal folds.	NR	IVIG, dantrolene, methylprednisolone.	After IVIG he continued with loose stools. The digestive evolution after corticosteroids was not reported.
**11**	Ishii K (2010) [16]	13 (13)	Male	NR	Anti-ACh receptor	Colonoscopy documented no abnormality.	Lymphocytic colitis.	Steroid and dantrolene.	Control of all his symptoms to achieve complete remission for 3 years.
**12**	Castiglioni C (2009) [2]	9 (10)	Female	D-xylose positive.	Anti-endomysial antibody negative.	Upper digestive endoscopy: leucoplakias were observed in the duodenum and a pronounced vasculature.	Moderate increase of the lymphoplasmacytic infiltrates of the lamina propria with granular neutrophils and eosinophils.	Prednisone. Restriction of simple carbohydrates from the diet.	Improvement of diarrhea with corticosteroids and disappearance after restriction of simple carbohydrates.
**13**	Asherson RA (2008) [5]	52 (53–54)	Female	NR	Anti-endomysial and anti-gluten antibodies both negative. ANA (1/1280). Anti-microsomal and anti-thyroglobulin antibodies.	The patient underwent foregut and hindgut endoscopy, but no significant abnormalities were detected. Later, upper endoscopic study with infiltration of the duodenum.	Biopsy of the infiltrated area compatible with eosinophilic enteritis.	Steroids, IVIG, diazepam, cyclophosphamide, azathioprine.	NR
**14**	Matsuura E (2007) [6]	8 (15)	Female	D-xylose positive.	Band of 90-kDa position on western blot for brain, spinal cord, stomach and duodenum tissue lysates.	Upper gastrointestinal tract: almost the entire mucosa of the stomach was atrophic, and multiple ulcer scars were observed, mainly in the body. Small white granules -speculated to be a type of secretion- were observed from the first to the second portion of the duodenum.	Histopathology of the first portion of the duodenum showed mucosal infiltration with inflammatory cells. *Helicobacter pylori* was not present.	NR	NR
**15**	Heger S (2006) [17]	12	Female	D-xylose positive.	ANA (1/640)	Endoscopy of the oesophagus, stomach, duodenum and ileo-colon with small duodenal ulcerations.	NR	Carbamazepine, IVIG, corticosteroids, methotrexate.	Diarrhea improved after treatment with methotrexate.
**16**	Nagahama T (2006) [4]	17 (21)	Female	D-xylose positive. Fecal clearance of α1antitripsina positive. Oral glucose tolerance test with 75 g glucose revealed a flat curve.	ANA	Upper-GI endoscopy revealed a normal esophagus and numerous nodular protrusions, akin to submucosal tumors, involving the cardia and the body of the stomach. Kerckring’s folds in the duodenum were lost, and the mucosa showed a fine granular appearance with white spots; a portion of the mucosa also showed small polypoid protrusions. Total colonoscopy revealed only an occasional ulcer scar in the cecum, the descending colon, and the sigmoid colon.	Histopatology of biopsy specimens from the stomach, the duodenum, and the large intestine did not provide any definitive diagnosis, because the findings were not characteristic. Autopsy data available.	Total intravenous hyperalimentation.	Death
**17**	Ezgu FS (2005) [18]	11 (11)	Female	NR	NR	NR	NR	Prednisolone	After three months, diarrhea disappeared.
**18**	Ashalatha R (2004) [19]	10 (10)	Female	NR	NR	Upper gastrointestinal endoscopy: normal.	Biopsy normal.	Phenytoin and prednisolone (previously clonazepam, tetrabenazine, baclofen, and diazepam).	NR
**19**	Cecchin CR (2003) [20]	7	Female	D-xylose normal.	NR	Upper gastrointestinal endoscopy showed a 2-cm duodenal erosion.	Gastric and duodenal biopsies showed chronic gastritis and duodenal ulcer, respectively.	Prednisone and amitriptyline.	Asymptomatic (diarrhea was not named).
**20**	Oyama M (1999) [21]	4 (13)	Female	NR	ANA (1/640)	They only stated that gastrointestinal tract examination was normal, with no inflammatory findings (endoscopy?).	NR	Antispasmodic and antipyretic drugs at first. Prednisolone.	Antispasmodic and antipyretic drugs caused only a decrease in the muscle spasms and diarrhea. Not reported after starting steroids.
**21**	Ikeda K (1998) [8]	30 (30)	Female	NR	ANA (×40) anti-microsomal and anti-thyroid antibodies.	NR	NR	Dantrolene and prednisolone.	NR
**22**	Merello M (1994) [22]	36	Male	NR	NR	NR	NR	Dantrolene. Phenytoin and carbamazepine, botulinum toxin.	NR
**23**	Ikegawa S (1993) [23]	4 (14)	Female	NR	NR	NR	NR	Dantrolene	NR
**24**	Kuru S (1992) [24]	12 (12)	Male	D-xylose normal.	NR	NR	NR	Several muscle relaxants. Corticosteroids.	NR
**25**	Yamagata T (1991) [25]	13	Female	Slight alteration in carbohydrate absorption.	ANA	NR	NR	Glucocorticoids	Free of symptoms at 6 months, but no other reference.
**26**	De-Xin W (1985) [26]	13 (13.5)	Female	Oral glucose tolerance test showed a relatively flat curve.	NR	NR	NR	Carbamazepine, phenobarbital, quinine sulfate and chlorpromazine	NR
**27**		9	Female	Oral glucose tolerance test showed a flat curve.	NR	Fibrogastroscopy with mucosal atrophy of the stomach and duodenum. A fungoid lesion of 0.3 mm diameter was seen on the mucosal surface of the stomach.	NR	Neostigmine and Chinese traditional medicine.	NR
**28**	Averianov IN (1984) [27]	10 (11)	Female	D-xylose positive.	NR	Colonoscopy: normal.	NR	Thioridazine, haloperidol, phenytoin, acetazolamide.	NR
**29**	Satoh A (1983) [28]	19 (21)	Female	D-xylosa positive. Oral glucose tolerance test showed a flat curve.	Anti-ACh receptor antibody.	NR	NR	Dantrolene	NR
**30**	Itahara K (1976) [29]	7 (8)	Female	Oral glucose tolerance test showed a flat curve.	NR	Gastroscopy: “Mosaic pave with stone” and partially “small polyp-like” edematous mucosa at corpus ventriculi, pars pylorica and duodenum.	Normal and/or atrophic chief cells in mucosa with edematous submucosa tissue or lymphangiectasia.	NR	NR
**31**	Inoue K (1976) [30]	NR (21)	Female	Oral glucose tolerance test showed a flat curve.	NR	Disseminated white spots in the jejunum.	Fat droplets in epithelial cells after administration of olive oil by tube.	NR	NR
**32**	Satoyoshi E (1975) [1]	9 (10)	Female	D-xylosa positive. High fecal excretion of I-triolein. Altered oral glucose test.	NR	NR	Autopsy data available. Gastroenteritis cystica polyposa.	NR	Death
**33**		9 (9)	Male	D-xylosa positive.	NR	NR	NR	NR	NR
**34**		14?	Female	D-xylosa positive. High fecal excretion of I-triolein. Oral glucose tolerance test showed a flat curve.	NR	NR	Autopsy data available. Gastroenteritis cystica polyposa.	NR	Death
**35**		12 (16)	Female	NR	NR	NR	NR	NR	NR
**36**		7 (16)	Male	NR	NR	NR	NR	NR	NR
**37**		7 (10)	Female	NR	NR	NR	NR	NR	Two years later, frequent defecation unchanged.
**38**		9 (9)	Male	Malabsorption was suspected, but test were not performed.	NR	NR	NR	NR	Suicide at age 19.
**39**		10 (30)	Female	NR	NR	NR	NR	NR	NR

*ANA* Anti-nuclear antibodies; *Anti-ACh receptor* Anti acetyl-choline receptor; *Anti-GAD* Anti-Glutamate acid decarboxylase; *IVIG* Intravenous immunoglobulins; *NR* Not reported

#### Inclusion criteria

The cases were considered to have gastrointestinal manifestations if at least one of the following circumstances was present:

- Diarrhea, defined as an increase in the frequency of bowel movements, accompanied by a decrease in their consistency. The presence of abdominal pain was considered when it was not related to the presence of spasms or cramps in the abdominal musculature.
- Positive malabsorption test results, including a positive D-xylose test or other evidence of carbohydrate malabsorption. Data compatible with protein or iron absorption deficit have also been considered.
  -Histological alterations in any section of the digestive tract. Histological samples were obtained by endoscopy or necropsy.
- Endoscopic and radiological data consistent with digestive involvement or malabsorption, such as atrophy or loss of intestinal folds.

#### Data extraction

The following data were extracted from each of the cases:

- Clinical and epidemiological data: age of onset of any of the symptoms pertaining to the syndrome, age of onset of digestive symptoms, sex and country of origin. Presence of diarrhea, number of bowel movements, abdominal pain, bloating, nausea, vomiting, weight loss, short stature or stunted growth, and symptoms of malnutrition or cachexia.
- Analytical data: the presence of anemia, iron deficiency, hypoproteinemia or hypoalbuminemia. Presence of autoantibodies.
- Malabsorption tests: results of the D-xylose Test and other malabsorption tests.
- Results of simple radiology tests, ultrasound, computed tomography, barium intestinal transit, or enema.
- Results of endoscopic examinations: gastroscopy or colonoscopy.
- Histological data obtained through endoscopy, biopsy, and from samples collected during surgery or necropsy.
- Treatments used, evolution and prognosis of digestive symptoms, and data regarding analytical or histological alterations, if available. Morbimortality and sequelae.

## Results

### Epidemiological data

The mean age of presentation of SS was 12.8 years (range, from a few months to 52 years), and the mean age of presentation of digestive symptoms was slightly higher (15.08 years, range from a few months to 53 years). Thirty-two patients (82%) were under 18 years of age at onset of disease. In 10 cases, the digestive symptoms were part of the manifestation of disease onset [1, 2, 7, 8, 13, 15, 19, 31], together with muscular spasms. Thirty-two of the patients were women (82%). Eighteen of these 39 cases (46.15%) were Japanese patients, although cases have been described worldwide.

### Gastrointestinal clinical features

The most common symptom was diarrhea, present in 36 (92.3%) cases [1, 2, 4–8, 11–16, 18–32]. Diarrhea was described as an increase in the number of stools and a decrease in consistency. The diarrhea of patients with SS is of a chronic nature, although not necessarily continuous; in fact, it may present as recurrent episodes [13, 22]. The number of daily stools was between 2 [27] and10 [2]. It was reported that foods with high carbohydrate content could trigger an episode of diarrhea [19]. Moreover, low carbohydrate diets were said to improve diarrhea [2]. Mild steatorrhea was also reported in one case [28]. There were no reports of blood, mucus or pus in the stool.

Other digestive symptoms associated with diarrhea were recurrent abdominal pain (6 cases) [2, 5, 7, 14, 20, 21], nausea (2 cases) [5, 20] and vomiting (2 cases) [15, 22]. Weight loss was reported in 9 patients [1, 4, 5, 11, 16, 22], and short stature or growth retardation in children were reported in 16 cases [1, 2, 6, 11, 14, 17–19, 23, 26, 27, 29]. Severe diarrhea with cachexia and dehydration could have led to the deaths of 3 patients [1, 4].

### Laboratory analyses

In 26 patients, results of laboratory analyses were provided, such as hemograms or basic biochemistry data [1, 2, 4–6, 8, 11, 13–17, 19–21, 23–30]. Thirteen patients reported anemia [1, 4–6, 11, 19, 23, 26, 28–30], which was usually microcytic, but hemoglobin levels were only reported in seven patients, and ranged between 8.5 and 12.1 mg/dl [4–6, 8, 26, 28, 30]. In 6 out of 10 cases, there was an explicit mention of iron deficiency [5, 6, 11, 14, 19, 30]. In 7 [1, 4, 6, 8, 23, 24, 29] out of 11 patients [1, 4, 6, 8, 14, 15, 23, 24, 27, 29, 30], hypoproteinemia was described, and in only one [8] of 4 patients [8, 13, 15, 27], hypoalbuminemia was present. Total cholesterol was reported in 11 cases, and its level ranged between 98 mg/dL and 169 mg/dL [1, 4–6, 24, 25, 27, 28, 30]. In 6 patients, total triglycerides were reported [5, 6, 8, 25, 27, 28], which ranged between 54 and 115 mg/dL. Transaminase levels were normal in all 14 patients for whom they were reported [1, 2, 4, 5, 13, 17, 20, 24–28, 30].

Only one case presented fecal occult blood [24]. In another case, there was no increase in eosinophils or presence of eggs or parasites in feces in the only case in which these parameters were assessed [16].

Carbohydrate malabsorption was assessed by oral glucose tolerance in the first cases published, being progressively replaced in more recent studies by the more accurate D-xylose test. The D-xylose test detected carbohydrate malabsorption in ten out of twelve cases [1, 2, 4, 6, 11, 17, 27, 28]. In one case with a positive D-xylose test, diarrhea was not reported [17]. Two cases of diarrhea with a negative D-xylose test were also reported [20, 24]. In 13 patients, an oral glucose tolerance test with 50 to 100 g of glucose was performed [1, 4, 8, 17, 19, 24, 26, 28–30]. One patient was unable to complete the test due to intolerance [19]. The glucose curve showed a flattened morphology in 9 of the remaining 12 cases, as a manifestation of carbohydrate malabsorption [1, 4, 26, 28–30]. In five of these nine patients, both tests (D-xylose test and oral glucose tolerance test) were positive [1, 4, 28]. There was a case with a negative oral glucose tolerance test and a positive D-xylose test [17]. Protein loss through the gastrointestinal tract, measured by alpha-1 antitrypsin clearance, was also described in one patient [4]. This patient also suffered from carbohydrate malabsorption, as demonstrated by both the D-xylose test and the oral glucose tolerance test. The I-131-triolein test in feces for fat malabsorption yielded pathological results in two patients [1]. Two cases with increased fat in stool [1] and mild steatorrhea [28] were also found.

Antinuclear antibody determination was positive in 8 [4, 5, 7, 8, 17, 21, 25] out of 16 cases [4, 5, 7, 8, 11, 13–17, 19–21, 24, 25]. The title oscillated between 1/40 and 1/1280 [5, 7, 8, 17, 21]. In three cases, the pattern of antinuclear antibodies was speckled [5, 7, 25]; one of these cases had SSB antibodies [7]. Other autoantibody tests for SSA, anti-RNP and anti-SM were performed in 4 patients [6, 11, 21, 25], but the results were negative. There was no reference to the antibody pattern in the remaining five patients. In one patient, anti-gliadin antibodies were detected [14]. In two other cases, anti-endomysial antibodies were negative [2, 5]. No anti-transglutaminase antibodies were reported in any patient. Anti-acetylcholine receptor antibodies were observed in four [11, 13, 16, 28] out of eight patients [6, 8, 11, 13–16, 28]. Other autoantibodies detected included: anti-thyroid antibodies in two [5, 8] out of 6 patients [5, 8, 13, 15, 17, 28], anti-GAD in one [11] out of 5 patients [5, 11, 14–16], anti-histone antibodies in one case [7], and reactivity against unknown neuronal antigens in another one [13]. Endo et al. [38] detected serum autoantibodies on brain tissue lysates using the western blot technique in a SS patient. Matsuura et al. described the case of a patient with SS and diarrhea, for whom a western blot was performed by incubating patient serum with different tissue lysates. They detected a band at the 90-kDa position for brain, stomach and duodenum tissue lysates, but not for spinal cord and uterine tissue lysates. Furthermore, the authors found the same autoantibodies in another patient in this study [6]. We have also recently confirmed the presence of those same autoantibodies detected by Matsuura et al. in the serum of a Spanish patient [11, 33, 34], using western blot for human brain, stomach and duodenum tissue lysates (unpublished data). Moreover, Aghoram et al. detected nonspecific immune reactivity in a patient’s serum exposed to monkey cerebellum and peripheral nerve tissue using indirect immunoflourescence [13].

### Radiological features

Abdominal radiological tests were described in 9 cases [1, 4, 6, 13, 15, 19, 27] (Table 1). In only two cases, do the authors comment on simple radiology studies. Nagahama described radiological findings in the small intestine, with loss of Kerckring’s folds throughout [4]. Ashalatha described abdominal ultrasound and abdominal CT, without pathological findings in the digestive tract [19]. Aghoram also reported a normal abdominal CT in a 30 year-old man with SS [13].

Five patients underwent oral barium studies [1, 6, 27] and in two cases, had barium enemas [1]. Aver’ianov reported a 13-year old girl who had a normal oral barium study [27]. Satoyoshi reported 3 cases in which oral barium studies were normal in the early stages; however, in later stages, two of these three cases yielded abnormal barium results [1]. This same author reported two more patients with barium enemas that were normal in the early stages, but abnormal in one of the patients at a later stage [1]. Matsuura, with a double contrast oral barium study, found a mild luminal dilatation and a decrease of Kerckring’s folds, which can be interpreted as secondary to chronic inflammatory damage. Matsuura et al. also reported a fine granulation of the second portion of the duodenum and “mesh-like” changes in the mucosa at the end of the second and third portions of the duodenum. An unclear contour and flocculation of the barium was also described, suggesting a malabsorption syndrome [6].

Finally, Ishii performed scintigraphy with a 99mTc-highsolid anaerobic digestion pool in a patient with Satoyoshi that showed an accumulation of isotopes in the colon and small intestine, suggesting leakage of albumin [16].

### Endoscopic findings

Sixteen patients underwent digestive endoscopy [2, 4–6, 8, 13, 15–17, 19–21, 26, 27, 29, 30]. Of these, 13 patients received an upper digestive endoscopy [2, 4–6, 8, 15, 17, 19–21, 26, 29, 30] and in 8 cases, a colonoscopy [4, 5, 8, 15, 13, 16, 17, 27]. In one patient, two upper gastrointestinal endoscopies were performed. The main endoscopic findings are reported in Table 1.

In one case, the endoscopy was macroscopically normal, although histological alterations were found [16]. In another patient, the endoscopic abnormal findings were discovered following a second endoscopic study [5]. Endoscopic findings included: atrophy of the stomach mucosa, with multiple ulcer scars mainly in the gastric body together with whitish granules from the first to the second portion of the duodenum [6], gastric or duodenal ulcerations [6, 17, 20], duodenum leukoplakia [2] and areas of duodenal mucosal infiltration [5]. Colonoscopy findings were: inflammation [13], flattening of mucous folds [15] and ulcerations [4]. Five colonoscopies were normal [5, 8, 16, 17, 27]. A characteristic endoscopic finding described by Nagahama et al. was the presence of nodular protrusions similar to submucosal tumors that can affect different parts of the digestive tract, including the cardia, body of the stomach and duodenum [4]. Using endoscopic ultrasonography, these protrusions appeared as cystic lesions in the stomach wall [4] compatible with the lesions of gastritis cystic polyposa, the histology of which is described below.

### Histological features

Data on the histology of the gastrointestinal lesions were described for 11 patients [1, 2, 4–6, 13, 16, 19, 20, 29] (Table 1). In three of these cases [1, 4], the histological description came from autopsy examination. In 7 patients, the samples were obtained during an upper digestive endoscopy [2, 4–6, 19, 20, 29]. In three cases, the data came from biopsies extracted during a colonoscopy [4, 13, 16]. In one patient, the histological findings were derived from colonoscopy, upper gastrointestinal endoscopy and autopsy [4].

-*Biopsies from upper gastrointestinal endoscopy*: There was a broad spectrum of histological manifestations, including normal biopsies [19] or unspecific findings [4]. The most frequent finding was an inflammatory infiltrate [2, 5, 6], predominantly lymphoplasmacytic [2], although eosinophilic infiltrate was also described [5]. Atrophy of chief cells and edematous submucosal tissue in the stomach was found in one case [29]. Findings compatible with chronic gastritis [20] were reported in one patient.
-*Colon biopsies*: Ishii et al. reported findings compatible with a diagnosis of lymphocytic colitis [16]. Aghoram et al. described findings suggestive of collagenous colitis [13].. Nagahama et al. reported that histopathologic examinations did not yield characteristic findings [4].
-*Autopsy findings*: Data from 3 autopsies were described. Satoyoshi reported two cases of SS with autopsy data [1], and Nagahama et al. described another one [4]. The average time elapsed between the age of symptom onset and the time of necropsy in these three patients was 18.6 years. In the rest of the patients, the time from onset of symptoms to biopsy was 7.7 years. Autopsy data revealed alterations throughout the gastrointestinal tract. Infiltration of gastric and duodenal mucosa by predominantly lymphoplasmacytic cells was described in all cases [1, 4]. This infiltration of the mucosa also affected the small intestine and the colon. Together with the inflammatory infiltrate, fibrosis of the mucosa and thickening of the muscularis mucosa were present. A characteristic finding in all three cases was the appearance of cystic lesions throughout the gastrointestinal tract, corresponding to glandular cystic dilations containing PAS-positive material. Ulcerative lesions were also found, penetrating the muscularis mucosa in the rectum [1].

### Treatment and outcome

Data regarding treatment were present in 28 of the cases with digestive symptoms [2, 4, 5, 7, 8, 11–28, 31, 32]. However, outcome in terms of gastrointestinal symptoms was described in only 12 [1, 2, 4, 11–18, 21].

In one patient, the treatment administered was hyperalimentation iv, but diarrhea did not improve [4]. In another patient, different treatments were prescribed, including phenytoin, carbamazepine, immunoglobulins and dantrolene, but likewise, diarrhea did not improve [15]. After that, this patient began corticosteroid treatment, but there is no reference as to whether this measure improved loose stools [15]. One patient improved with carbamazepine and otilonium bromide treatment [14]. Oyama et al. reported that in their patient, antispasmodic and antipyretic drugs only caused a slight decrease in diarrhea; however, outcome after beginning corticosteroid therapy was not described [21].

In 6 patients, diarrhea improved with a treatment regimen that included corticosteroids, alone or in combination [2, 11–13, 16, 18]. In addition to these six patients, two other cases that were “asymptomatic” without express reference to digestive manifestations, had also received corticosteroid therapy [20, 25]. Treatment with corticosteroids or immunosuppressants can cause remission of the whole symptomatic spectrum of SS. However, in one of the patients that improved with corticosteroids, diarrhea improved further after including a low-carbohydrate diet in the treatment plan [2]. Another patient improved by adding methotrexate after corticosteroids failed to control diarrhea [17]. Complete remission of diarrhea for at least 3 years was reported with corticosteroid treatment [16].

Four patients died, three of them due to the poor evolution of the disease [1, 4]. Two of them had severe malnutrition and uncontrolled diarrhea. They developed calcium and ionic disturbances. Both patients died after an episode of seizures followed by coma [1]. A third patient was treated with intravenous hyperalimentation due to diarrhea and malabsorption to improve her nutritional status. She also had episodes of acute pancreatitis. One year later, she underwent surgery consisting of gastrojejunostomy, percutaneous enterostomy, and percutaneous cholangiostomy. A few months after surgery, the patient died of sepsis with multiple organ failure [4]. The fourth patient committed suicide ten years after the onset of the disease [1]. It is not reported that these patients received corticosteroids or immunosuppressants.

## Discussion

As demonstrated in our review, more than half of the patients described with SS have digestive manifestations, of which the main symptom is diarrhea. The clinical picture of these patients can vary from practically asymptomatic with a positive D-xylose test, to very symptomatic patients with more severe diarrhea and signs of malabsorption, cachexia and dehydration. Diarrhea in SS can lead to significant consequences for the patient, including malnutrition, growth retardation, interference with daily life activities and even death. Diarrhea in SS is usually non-inflammatory, without blood or mucus in the stool. The D-xylose test appears to be more accurate in determining carbohydrate malabsorption; therefore it has been replacing the oral glucose tolerance test used mainly in the first published cases.

Most of the radiological tests performed showed non-pathological or non-specific findings. Endoscopic explorations often revealed alterations in the gastrointestinal mucosa, such as infiltrative and granular mucosa or ulcers in stomach, duodenum and colon. Histological data showed a predominantly lymphoplasmacytic inflammatory infiltrate that can involve all sections of the digestive tract. In the 3 patients who underwent autopsy, cystic lesions suggestive of gastritis cystica polyposa were discovered. This variation in lesions, ranging from a mild inflammatory infiltrate in the mucosa to more severe lesions with atrophy, fibrosis and the appearance of cystic lesions, suggests a progressive disease course. Moreover, autopsy patients showed more severe lesions. The time elapsed between the age of symptom onset and necropsy/biopsy was greater (18.6 years) in these patients than for the other subjects (7.7 years).

The combination of diarrhea with muscle spasms, cramps, alopecia and osteoarticular deformities is very specific to SS [1]. However, SS may mimic other diseases with non-inflammatory diarrhea, weight loss, growth retardation and signs and symptoms of malabsorption, including: celiac disease, refractory sprue, tropical sprue, Whipple’s disease, drug-induced colitis, autoimmune enteropathy, amyloidosis or inflammatory bowel disease [39, 40].

In terms of the differential diagnosis (Table 2) of SS, celiac disease and SS have many overlapping symptoms. For example, they both present manifestations secondary to nutrient malabsorption, with diarrhea, weight loss or iron-deficiency anemia. Both diseases also predominate in women in the first decades of life. Additionally, celiac disease can be associated with other conditions described in SS patients, such as autoimmune thyroid disease or myasthenia gravis [40]; even the rare manifestations of celiac disease, such as infertility, arthralgia, arthropathy or alopecia are part of the clinical spectrum of SS [40]. However, the improvement of symptoms with a gluten-free diet favors a celiac disease diagnosis, although this diet has not been tested in patients with SS. On the other hand, the appearance of muscle cramps points towards the diagnosis of SS. In terms of laboratory analysis, anti-gliadin antibodies present in celiac disease have been detected in one case of SS. Anti-endomysial antibodies also common to celiac disease were assessed in two SS cases, but with negative results. Anti-transglutaminase antibodies have not yet been tested for in SS. Histologically, the inflammatory cells in intestinal biopsies characteristic of celiac disease are also common to SS and may lead to an initial diagnosis of celiac disease in SS patients. In the future, detailed characterization of intraepithelial lymphocytes may provide important distinguishing features for Satoyoshi patients.

**Table 2:** Differential diagnosis of diarrhea caused by Satoyoshi syndrome

	Similarities with Satoyoshi syndrome	Differences with Satoyoshi syndrome
**Celiac sprue**	Most frequent in women in the first decades of life. Diarrhea with nutrient malabsorption Immune basis Iron deficiency anemia Weight loss Associated with other conditions such as autoimmune thyroid disease or myasthenia gravis. It can associate with rare manifestations such as infertility, arthralgia, arthropathy or alopecia. Intestinal biopsies with inflammatory infiltrate	Improvement with gluten free diet. Muscle spasm or cramps not present
**Drug induced Diarrhea**	Diarrhea Duodenitis with inflammatory infiltrate in biopsies	Improvement after drug withdrawal Muscle spasm and alopecia are not present
**Inflammatory bowel disease**	Diarrhea and weight loss It can be present in young patients	Inflammatory diarrhea, proctitis, bloody diarrhea, tenesmus, perianal fistulas, stenotic or fibrotic alterations osteoarticular involvement in form of migratory and asymmetric polyarthritis. In SS, bone deformities and alterations in the metaphysis are more frequent.
**Irritable bowel syndrome**	Increased frequency of bowel movements	No signs of malabsorption or malnutrition. Associated with fibromyalgia, chronic fatigue syndrome, chronic back pain, chronic pelvic pain, chronic headache, and temporomandibular joint dysfunction, depression, anxiety
**Autoimmune enteropathy**	Immune-mediated intestinal mucosal atrophy can be cause of watery diarrhea	Alopecia, muscle spasms and skeletal alterations are nor present in autoimmune enteropathy

As in SS, inflammatory bowel disease can also manifest in young patients with diarrhea and weight loss. However, signs of inflammatory diarrhea, proctitis, bloody diarrhea, tenesmus, perianal fistulas and stenotic or fibrotic alterations do not occur in patients with SS. Arthritis can be present in inflammatory bowel disease, mainly in Crohn’s disease in the form of migratory and asymmetric polyarthritis; in SS, however, the most frequent osteoarticular manifestations are bone deformities and alterations in the metaphysis, but not arthritis.

With respect to irritable bowel syndrome, there may be increased frequency of bowel movements, but this syndrome usually involves abdominal pain and distension, which are less frequent in SS. In addition, irritable bowel syndrome does not show signs or symptoms related to the malabsorption, weight loss or malnutrition of SS.

Autoimmune enteropathy, a rare condition that can occur both in children and adults, must also be differentiated from SS. It is characterized by immune-mediated intestinal mucosal atrophy that can affect the esophagus, stomach, small bowel and colon. However, it is the clinical picture of SS, with alopecia, muscle spasms and skeletal alterations that differentiates it from autoimmune enteropathy.

Medications, such as nonsteroidal anti-inflammatory drugs (NSAID) or olmesartan, can also cause diarrhea and duodenitis. NSAID duodenitis presents with histological alterations in the duodenum, reminiscent of those of celiac disease. These lesions consist of a non-specific infiltration of the neutrophil and plasma cells in lamina propria, with intraepithelial lymphocytes and low-grade villous blunting. Furthermore, prolonged use of these anti-inflammatory drugs can cause iron-deficiency anemia. On the other hand, in some patients, olmesartan can induce severe diarrhea associated with duodenal inflammation indistinguishable from other pathologies, such as celiac disease. Improvement after drug withdrawal, as well as the other manifestations of SS, can help in the differential diagnosis. The use of these drugs is not described in the patients included in this review.

The treatment of diarrhea does not differ from that of the other clinical manifestations in SS. Corticosteroids and immunosuppressants remain the treatment of choice for diarrhea in patients with SS. Although there is not much data in the follow-up of these patients, at least one case has been documented in which improvement was maintained throughout 3 years of follow-up [16].

The main limitation of our review is that it is composed of isolated cases. Besides, symptom descriptions lack detail. The authors refer to the presence of diarrhea in the majority of cases; however, this symptom is poorly characterized. On the other hand, the most complete histological data come from autopsies published many years ago and performed on SS patients who were not treated with immunosuppressants.

Findings such as the presence of specific antibodies against brain, stomach and intestinal lysates have been reported by two authors [6, 38]. These findings are consistent with the autoimmune character of SS and could be useful in the future as a diagnostic tool. More studies are needed to clarify the role of these antibodies in both the pathogenesis of the syndrome and the diagnostic approach to patients.

## Conclusions

Chronic diarrhea with malabsorption is one of the most disabling symptoms in SS. The clinical spectrum of these patients can vary from practically asymptomatic with a positive D-xylose test, to very symptomatic patients with severe diarrhea. Thus, malabsorption testing seems advisable in presumed SS patients, even if digestive symptoms are absent. Corticosteroids and immunosuppressants are still the treatment of choice for diarrhea in these patients. SS shares clinical manifestations with celiac disease and other autoimmune and gastrointestinal inflammatory syndromes, thus differential diagnosis is key. The recognition of SS is essential for early immunosuppressive treatment and for achieving a favorable outcome. In this regard, determining the specific auto-antibody pattern for SS remains a pending issue for future research in this field.

## Data Availability

Not applicable.

## Abbreviations

ANA: Anti-nuclear antibodies
Anti-ACh receptor: Anti acetyl-choline receptor
Anti-GAD: Anti-glutamate acid decarboxylase
IVIG: Intravenous immunoglobulins
NR: Not reported
SS: Satoyoshi syndrome
OMIM: Online mendelian inheritance in man
